# B-lactams serum concentrations in critically ill cirrhotic patients: a matched-control study

**DOI:** 10.1186/2197-425X-3-S1-A401

**Published:** 2015-10-01

**Authors:** O Lheureux, E Trepo, M Hites, F Cotton, F Wolff, R Surin, J Creteur, J-L Vincent, T Gustot, F Jacobs, FS Taccone

**Affiliations:** ULB, Intensive Care, Brussels, Belgium; ULB, Gastroenterology, Brussels, Belgium; ULB, Infectious Disease, Brussels, Belgium; ULB, Clinical Chemistry, Brussels, Belgium

## Introduction

Few data are available on the pharmacokinetics (PKs) of b-lactams in critically ill cirrhotic patients.

## Objectives

*T*he objective of this study was to evaluate whether cirrhosis was associated with alterations in b-lactam concentrations when compared to other critically ill patients, and to identify the principal risk factors for inadequate concentrations in these patients.

## Methods

We reviewed data from critically ill cirrhotic patients and matched controls in whom routine therapeutic drug monitoring (TDM) of broad-spectrum β-lactam antibiotics (ceftazidime or cefepime, CEF; piperacillin/tazobactam; TZP; meropenem, MEM) was performed. Serum drug concentrations were measured twice during the elimination phase by high-performance liquid chromatography (HPLC-UV). Antibiotic PKs were calculated using a one-compartment model. We considered therapy was adequate when serum drug concentrations were between 4 and 8 times the minimal inhibitory concentration (MIC) of *Pseudomonas aeruginosa* during optimal periods of time for each drug (≥70% for CEF; ≥ 50% for TZP; ≥ 40% for MEM).

## Results

We studied 42 cirrhotic patients (4 for CEF, 16 for TZP and 22 for MEM) and 42 matched controls. Drug dosing was similar in the two groups. The PK analysis showed a lower volume of distribution (Vd) of MEM (p = 0.05) and a lower antibiotic clearance (CL) of TZP (p = 0.009) in patients with cirrhosis when compared to non-cirrhotic patients. More cirrhotic patients have excessive serum b-lactam concentration (p = 0.015), in particular for TZP.

## Conclusions

Standard regimens of β-lactam resulted in excessive serum concentration in two-third of the patients in the cirrhotic cohort. These findings are mainly marked in cirrhotic patients treated by TZP, probably because of reduced drug CL.

